# Eukaryotic Translation Initiation Factor 3 Subunit B Is a Promoter in the Development and Progression of Pancreatic Cancer

**DOI:** 10.3389/fonc.2021.644156

**Published:** 2021-04-29

**Authors:** Haoyuan Ren, Gang Mai, Yong Liu, Rongchao Xiang, Chong Yang, Wenjie Su

**Affiliations:** ^1^Department of Gastrointestinal Surgery, People's Hospital of Deyang City, Deyang, China; ^2^Organ Transplantation Center, Sichuan Provincial People's Hospital, University of Electronic Science and Technology of China, Chengdu, China; ^3^Department of Anesthesiology, Sichuan Provincial People's Hospital, University of Electronic Science and Technology of China, Chengdu, China; ^4^Chinese Academy of Sciences Sichuan Translational Medicine Research Hospital, Chengdu, China

**Keywords:** pancreatic cancer, EIF3B, cell proliferation, cell migration, cell apoptosis

## Abstract

**Background:** Pancreatic cancer (PC) is a malignant tumor with hidden incidence, high degree of malignancy, rapid disease progression, and poor prognosis. Eukaryotic translation initiation factor 3 subunit B (EIF3B) is necessary for tumor growth, which is an alternative therapeutic target for many cancers. However, little is known about the relationship between EIF3B and PC.

**Methods:** The expression of EIF3B in PC was detected by immunohistochemistry. EIF3B knockdown cell models were constructed by lentivirus infection. The MTT assay, the wound-healing assay, the transwell assay, the flow cytometry, and the Human Apoptosis Antibody Array was used to detect the effects of EIF3B knockdown on cell proliferation, cell migration, cell apoptosis, and cell cycle *in vitro*. Also, the effects of EIF3B knockdown on the tumor growth of PC were determined *in vivo*.

**Results:** This study showed that the expression level of EIF3B was significantly up-regulated in PC tumor tissues and associated with pathological grade. *In vitro*, EIF3B knockdown inhibited the PC cell proliferation and migration, and the apoptosis levels were obviously promoted by regulating apoptosis-related proteins including Bcl-2, HSP27, HSP60, Survivin, sTNF-R2, TNF-α, TNF-β, TRAILR-3, TRAILR-4, and XIAP. Furthermore, the tumor growth of PC was inhibited after the knockdown of EIF3B *in vivo*.

**Conclusion:** EIF3B was up-regulated in PC and was a promoter in the development and progression of PC, which could be considered as a therapeutic target for the treatment of PC.

## Introduction

Pancreatic cancer (PC) is a malignant tumor of the digestive system, which has the characteristics of hidden incidence, high degree of malignancy, rapid disease progression, poor prognosis, and high mortality rate (1, 2). The main reason for the high mortality rate of PC is the difficulty of early clinical diagnosis and the lack of effective clinical treatment. In addition, the mechanism of the development of PC is not fully understood (3). At present, the existing treatment methods for PC patients mainly include surgical resection and combined radiotherapy and chemotherapy. However, there are certain limitations. For example, the sectional progression and the tolerances to radiotherapy and chemotherapy occur in some patients after the treatment (4). Compared with traditional treatment methods, targeted drugs have the advantages of reaching tumor cells or tissues accurately (5, 6). With the progress of PC research and the continuous improvement of treatment methods, more new potential therapeutic targets have been discovered, and clinical application of a variety of specific therapeutic target inhibitors has achieved certain results (7, 8). For example, Moore et al. reported that erlotinib and gemcitabine could improve the survival of patients with advanced PC to a certain extent (9). However, some therapeutic target inhibitors in a clinical test did not obtain satisfactory results. For example, mTOR inhibitors and TRK inhibitors were used to evaluate the effects on the metastasis of PC cell, the results showed that the adverse reactions were well-tolerated, but the clinical efficacies were not obvious (10). Moreover, many clinical studies of inhibitors such as demcizumab, tarextumab, and RO4929097 obtained negative results (11, 12). Thus, the current targeted treatment has not yet brought a satisfactory improvement in the treatment of PC. It is of great significance to develop more specific and effective targets for the targeted treatment of PC.

As we know, the increase in protein synthesis promotes the proliferation of tumor cells, and sometimes, this proliferation is uncontrollable. At present, more research focused on the inhibition of translation factors regulating protein synthesis and activity, which hold the key to the initiation and progression of tumor and tumor prognosis (13). In the process of protein synthesis, eukaryotic translation initiation factors (EIFs) assemble 80S ribosomes and the initiator methionyl-tRNA (Met-tRNA) onto mRNA. EIFs with a total molecular mass of 700 kDa are composed of 13 subunits (EIF3A to M), which play a functional role in the initiation of cell and virus translation and cancer development (14, 15). EIF 3 subunit B (EIF3B), a major scaffold protein in EIFs, is associated with protein synthesis by interacting with EIF3A, G, I, and J (16). The human EIF3B is a protein composed of 814-amino acid with an RNA recognition motif (RRM) at its N terminus (17). Previous studies showed that EIF3B was abundantly expressed in colon cancer tissues, and the silencing of EIF3B inhibited the proliferation of colon cancer cells (18). In addition, EIF3B promoted the proliferation of glioblastoma tumor cells and might be related to the development of human glioblastoma (19, 20). Although the effects of EIF3B on other cancers have been analyzed, whether EIF3B played a role in human PC has not been explored yet.

In this study, we explored the effects of EIF3B on PC *in vitro* and *in vivo*. The expression of EIF3B was significantly up-regulated in tumor tissues and PC cell lines. EIF3B knockdown inhibited the abilities of proliferation and migration of PC cells, promoted cell apoptosis *in vitro*, and suppressed the tumor growth *in vivo*. In addition, the mechanism of the effects of EIF3B on PC was initially explored. EIF3B was related to the occurrence and development of PC and might be used as a potential therapeutic target for the treatment of PC.

## Materials and Methods

### Clinical Samples

PC tumor tissues and para-carcinoma tissues (tissues from tumor adjacent sections) were purchased from Shanghai Outdo Biotech Co., Ltd (HGla-Ade100PG01, Shanghai, China). There were 166 samples including 97 tumor tissues and 69 para-carcinoma tissues. All patients have signed informed forms before the collection of the tissue samples. The study was approved by the Medical Ethics Committee of People's Hospital of Deyang City.

### Cell Culture

The human PC cell lines PANC-1, SW1990, AsPC-1, and BxPC-3 were obtained from Cell Resource Center, Shanghai Academy of Life Sciences, Chinese Academy of Sciences. The cells were cultured in Dulbecco's modified Eagle's medium (DMEM) containing 10% fetal bovine serum (FBS) and maintained at 37°C in 5% CO_2_.

### Immunohistochemical Staining

The tissue slides were placed in the oven at 65°C for 30 min, then dewaxed in xylene, and washed in alcohol. The tissue slides were repaired by using 1 × EDTA solution (Beyotime Biotechnology Co., Ltd, Shanghai, China). After that, the slides were cooled to room temperature and then placed in 1 × phosphate-buffered saline (PBS) with Tween (PBST) buffer for several minutes. The slides were washed three times with 1 × PBST solution for 5 min each time, and the endogenous peroxidase of tissues was sealed with 3% H_2_O_2_ for 5 min. The slides were washed with 1× PBST solution according to previous steps. Finally, the slides were sealed with 5% serum for 15 min. The slides were incubated with primary antibodies (EIF3B: 1:100, Abcam, Cambridge, MA, USA) overnight at 4°C. Then, the sections were incubated with second antibody [goat anti-rabbit IgG H&L (HRP):1:400, Abcam, Cambridge, MA, USA] for 1 h at 37°C after being washed three times with 1 × PBST solution for 5 min each time. The slides were stained with DAB for 5 min, and then hematoxylin (Baso Diagnostics Inc., Zhuhai, China) was used for re-dyeing for 10–15 s. The slides were dehydrated by absolute ethanol and then dehydrated with alcohol and xylene for 2–3 min after washing with running water for 2–3 min. Finally, the slides were sealed with neutral resin (China National Pharmaceutical Group Co., Ltd, Beijing, China), and photographs were taken under a microscope. All slides were examined randomly by three independent pathologists. The degree of staining was evaluated as 0 (negative), 1 (weak), 2 (positive ++), and 3 (positive+ + +). The scores of positive cells were as follows: 0 (0%), 1 (1–25%), 2 (26–50%), 3 (51–75%), or 4 (76–100%). The samples were divided into four levels including negative (0), positive (1–4), ++ positive (5–8), and + + + positive (9–12); and the numbers in parentheses are the values of positive cell scores ^*^ staining color intensity scores.

In terms of Ki-67 immunostaining, the tumor tissues were fixed with formalin and then embedded with paraffin. Then all slides were blocked with PBS–H_2_O_2_. Next, the slides were incubated with primary antibody Ki-67 (1:200, Abcam) and secondary antibody goat anti-rabbit IgG H&L (HRP) (1:400, Abcam) overnight at 4°C, respectively. Finally, the slides were stained by hematoxylin and eosin (Baso, Zhuhai, Guangdong, China). Stained slides were photographed with a microscope and scored as described above.

### Lentivirus RNAi Construction and Infection

With the use of EIF3B as a template, three RNAi target sequences shEIF3B1, shEIF3B2, and shEIF3B3 (GGGAGAGAAATTCAAGCAAAT, GAGTGGGATATTCCAGAGAAA, and GAAGAAAGAGCGAGATGGACA, respectively) were designed to silence EIF3B. shCtrl (scramble sequence: TTCTCCGAACGTGTCACGT) was used as negative control. These sequences were inserted into BR-V-108 vector and then transformed into DH5α cells and cultured overnight at 37°C. The EndoFree Maxi plasmid kit (Tiangen, Beijing, China) was used to extract plasmids. 293T cells in logarithmic growth phase were transfected with the shRNA expression vector and packaging vector using Lipofectamine 2000 transfection reagent (Thermo Fisher Scientific, Waltham, MA, USA). After being cultured in DMEM with 10% FBS, the lentivirus was collected for cell transfection; 40 μl of 1 × 10^8^ TU/ml lentivirus (shCtrl and shEIF3B) was transfected into PANC-1 and SW1990 cells with ENI.S + Polybrene additives. All cells were cultured for 72 h, and the fluorescence inside the cells was observed under microscope to evaluate the infection efficiency.

### Real-Time Quantitative PCR

The PANC-1 and SW1990 cells infected by lentivirus were cultured for 72 h to express EIF3B shRNA (shEIF3B) and control shRNA (shCtrl). Total RNA was extracted according to TRIzol reagent (Sigma, St. Louis, MO, USA), the concentration and quality of which were determined by Nanodrop 2000/2000C spectrophotometer (Thermo Fisher Scientific, Waltham, MA, USA). Then, reverse transcription was performed to obtain cDNA by using the Promega M-MLV Kit (Promega Corporation, Madison, Wisconsin, USA). Real-time quantitative PCR system used 12 μl and was conducted with SYBR Green Mastermixs Kit (Vazyme, Nanjing, Jiangsu, China). The primers used in qPCR were as follows (5′-3′): the forward primer of EIF3B was 5′-CCTGAAGAGGATGGGAAGACA-3′, and the reverse primer of EIF3B was 5′-AAGAGGTTGACCCGGAATG-3′. The forward primer of GAPDH was 5′-TGACTTCAACAGCGACACCCA-3′, and the reverse primer of GAPDH was 5′-CACCCTGTTGCTGTAGCCAAA-3′. GAPDH served as internal reference. The expression level of each sample relative to the target gene of NC group was calculated by the 2^−ΔΔCt^ method.

### Western Blot

The PANC-1 and SW1990 cells infected with LV-shCtrl or LV-shEIF3B were washed with PBS buffer. After that, the cells were lysed in ice-cold radioimmunoprecipitation assay (RIPA) buffer (Millipore, Temecula, CA, USA). The protein concentration was determined by BCA Protein Assay Kit (HyClone-Pierce, Logan, UT, USA), and 10% sodium dodecyl sulfate–polyacrylamide gel electrophoresis (SDS-PAGE) (Invitrogen, Carlsbad, CA, USA) was used to separate 20-μg proteins and then transferred into polyvinylidene difluoride (PVDF) membranes. The PVDF membranes were blocked with a blocking solution [Tris-buffered saline with Tween (TBST) solution containing 5% skim milk] at room temperature for 1 h, which were incubated with primary antibodies at room temperature for 2 h. Then, the membranes were washed three times with TBST, 10 min each time. After that, the membranes were incubated with second antibodies at room temperature for 2 h. Similarly, the membranes were washed as previous steps. Chemiluminescence was analyzed by a chemiluminescence imager (Amersham, Chicago, IL, USA). The primary antibodies used in western blotting are as follows: EIF3B (1:1,000, Abcam, ab124778), GAPDH (1:3,000, Bioworld, AP0063), Akt (1:1,000, CST, 4685), p-Akt (1:500, R&D, AF887-sp), CCND1 (1:2,000, Abcam, 2978), CDK6 (1:1,000, Abcam, ab151247), and PIK3CA (1:1,000, Abcam, ab40776). The secondary antibody used in western blotting was goat anti-rabbit (1:3,000, Beyotime, A0208).

### MTT Assay

After being infected with LV-shCtrl or LV-shEIF3B, PANC-1 and SW1990 cells in logarithmic growth phase were digested, resuspended, and counted. Cells were cultured in 96-well plates at the cell density of 2,000 cells (100 μl/well) and counted for 5 days. The next day, 20 μl of MTT solution (5 mg/ml, Genview, Florida, USA) was added into each well before 4 h of culture termination. After 4 h, the culture medium was discarded and replaced with 100 μl of dimethyl sulfoxide (DMSO) solution (Shanghai Shiyi Chemical Reagent Co., Ltd, Shanghai, China). After the medium was oscillated for 2–5 min, the optical density (OD) value was detected at 490-nm wavelength with a microplate reader (Tecan Infinite, Männedorf, Zurich, Switzerland). Three repetitive wells were set in each group.

### Wound Healing Assay

The PANC-1 and SW1990 cells were collected after the infection of shEIF3B and shCtrl and placed in a 96-well plate (5 × 10^4^ cells/well). The cell layers were scratched and then washed two to three times with serum-free medium. The low-concentration serum medium (0.5% FBS) was added, and the cells were incubated in an incubator with 5% CO_2_ at 37°C. The images were captured by a microscope at 0, 8, 16, and 24 h. Each experiment included three sets of replicates, and the cell migration rate was calculated based on the scratch picture.

### Transwell Assay

The 3422 Corning Transwell Kit (Corning, NT, USA) was used to conduct the transwell assay. First, the upper chamber was placed in a 24-well plate with 100 μl of serum-free medium and incubated for 1–2 h. PANC-1 and SW1990 cells infected with shEIF3B and shCtrl in logarithmic growth phase were collected and counted, then resuspended with low-concentration serum medium, and incubated in the upper chamber (8 × 10^4^ cells/well). In addition, 600 μl medium supplemented with 30% FBS was added in the lower chamber. Then, the upper chamber was transferred to the lower chamber and incubated in an incubator for 40 h. Finally, cells were stained by adding 400 μl of Giemsa, and the migration ability of cells was analyzed. The experiment was repeated three times.

### Flow Cytometry for Cell Apoptosis and Cell Cycle

After infection, both PANC-1 and SW1990 cells were inoculated in six-well plates (2 ml/per well). When the cell confluence reached 85% (5 days after lentivirus infection), the cells were collected, digested, and washed with 4°C pre-cooled D-Hanks. Then, the cell pellet was washed once with 1× binding buffer, centrifuged at 1,300 rpm for 3 min, and collected. Finally, the cells were stained by adding 10 μl of Annexin V-APC (eBioscience, San Diego, CA, USA) without light, and the levels of apoptosis were measured by using FACSCalibur (BD Biosciences, San Jose, CA, USA). For cell cycle, PANC-1 and SW1990 cells were inoculated in 6-cm dishes with 5 ml per well. When the cell confluence reached 70%, the cells were collected and washed once with PBS. Then, the cells were centrifuged at 1,200 rpm for 5 min. Next, the cells were washed once with 4°C pre-cooled PBS and centrifuged at 1,500 rpm for 5 min. Finally, the cells were fixed with pre-cooled 70% ethanol for at least 1 h and stained with propidium iodide (PI). FACSCalibur (BD Biosciences, San Jose, CA, USA) was used to analyze the changes of cell cycle. The proportion of the cells in different phases including the G1, S, and G2 phases was calculated and compared. Each experiment was repeated three times.

### Human Apoptosis Antibody Array Analysis

The SW1990 cells were selected to carry out the Human Apoptosis Antibody Array (Abcam, Cambridge, MA, USA) to further detect the effects of EIF3B knockdown on the levels of apoptosis-related protein expression. The cells were diluted with 2 × Cell Lysis Buffer. The Handling Array membranes were washed with Wash Buffer II and incubated with cell lysates and Biotin-conjugated Anti-Cytokines overnight at 4°C; and the membranes were completely covered with sample or reagent during incubation. During the experiments, the membranes were not allowed to dry out. Finally, the signals were determined by chemiluminescence imaging system.

### The Construction of Nude Mouse Tumor Formation Model

Four-week-old female BALB-c nude mice were provided by Shanghai Lingchang Experimental Animals Co., Ltd (Shanghai, China). The animal experiments were approved by the Medical Ethics Committee of People's Hospital of Deyang City. Xenograft models were constructed using shCtrl and shEIF3B SW1990 cells. The shCtrl and shEIF3B SW1990 cells in logarithmic growth phase were digested by trypsin and suspended into cell suspension. Then, the cell suspension was resuspended by D-Hanks or PBS, and the density of the cells reached 2 × 10^7^ cells/ml. After that, 200 μl of cell suspension (4 × 10^6^ cells) was injected subcutaneously into mice (10 mice/each group). The Vernier caliper was used to measure five times the volume of tumor during the feeding period. After 38 days, the mice were sacrificed, the tumors were removed, and the weight of the tumors was measured. Before being sacrificed, 0.7% sodium pentobarbital at the dosage of 10 μl/g was injected intraperitoneally for several minutes, and the fluorescence was observed by the *in vivo* imaging system (IVIS Spectrum, Perkin Elmer). Tumor tissues were frozen in liquid nitrogen and stored at −80°C.

### Statistical Analysis

All data were processed by GraphPad Prism 6 (San Diego, CA, USA) and presented in the form of the mean ± SD. The sign test and the Mann–Whitney U were used to analyze the difference of EIF3B levels between tumor tissues and para-carcinoma tissues, and the significant relationship between the expression of EIF3B and the pathological characteristics of PC, respectively. The Pearson correlation analysis was used to assess the correlation between the levels of EIF3B and pathological grade of PC. The statistical analysis included the unpaired *t*-test and one-way ANOVA in this study, and *P* < 0.05 was considered to be significantly different. All the above cell experiments were performed in triplicate.

## Results

### The Expression of Eukaryotic Translation Initiation Factor 3 Subunit B Was Up-Regulated in Tumor Tissues

**Figure 1:**
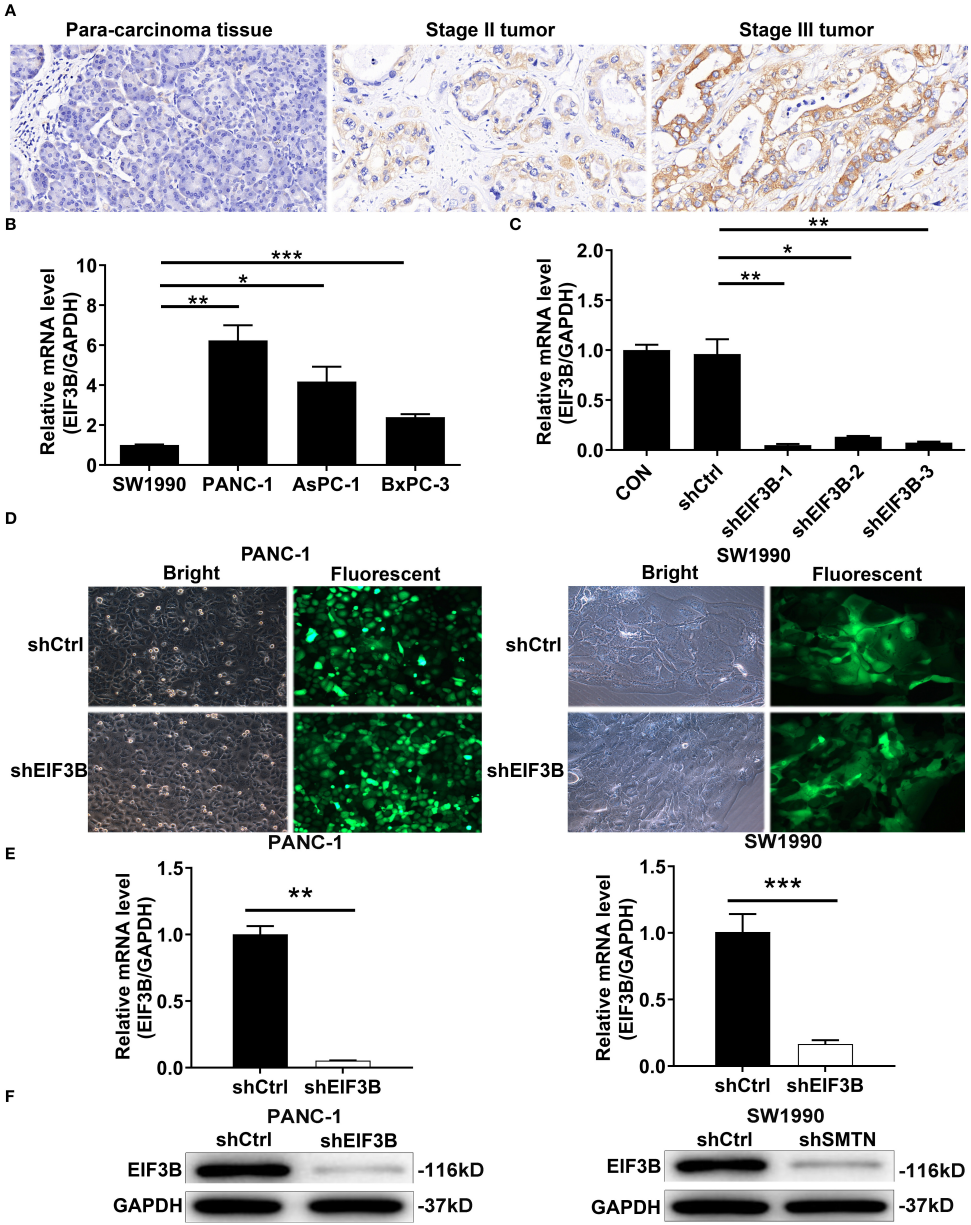
Eukaryotic translation initiation factor 3 subunit B (EIF3B) was up-regulated in pancreatic cancer (PC) and EIF3B knockdown cell model was constructed. **(A)** The expression levels of EIF3B in PC tumor tissues and para-carcinoma tissues were determined by immunohistochemical staining. Magnification times: 200 ×. **(B)** The expression of EIF3B in PANC-1, SW1990, AsPC-1, and BxPC-3 was detected by qRT-PCR. **(C)** The knockdown efficiencies of EIF3B in shEIF3B-1, shEIF3B-2, and shEIF3B-3 group were detected by qRT-PCR. **(D)** The fluorescence expression in cells was observed after 72-h infection. Magnification times: 200 ×. **(E)** The EIF3B expression in PC cell lines after infection was analyzed by qRT-PCR. **(F)** The expression of EIF3B protein in PC cell lines after infection was detected by western blot. Results are presented as mean ± SD. *P < 0.05, **P < 0.01, ***P < 0.001.

The expression levels of EIF3B in PC tumor tissues and para-carcinoma tissues were determined by immunohistochemical staining. It was obvious that EIF3B expression was significantly up-regulated in tumor tissues compared with para-carcinoma tissues (Figure 1A), which was also indicated by the statistical analysis (*P* < 0.001, Table 1). Furthermore, the Mann–Whitney U analysis was performed to explore the relationship between EIF3B expression and PC tumor characteristics, revealing a significant association between the expression of EIF3B and pathological grade (*P* < 0.01, Table 2). Moreover, the same results were obtained by the Spearman correlation analysis (*P* < 0.01, Table 3). Based on the above results, it could be seen that EIF3B might be related to the development of PC.

**Table 1:** The expression of EIF3B in pancreatic cancer tissues and para-carcinoma tissues revealed in immunohistochemistry analysis.

**EIF3B expression**	**Tumor tissue**		**Para-carcinoma tissue**		***P*-value**
	**Cases**	**Percentage**	**Cases**	**Percentage**	
Low	62	63.9%	41	59.4%	< 0.001
High	35	36.1%	28	40.6%	

**Table 2:** Relationship between EIF3B expression and tumor characteristics in patients with pancreatic cancer.

**Features**	**No. of patients**	**EIF3B expression**		***P-*value**
		**Low**	**High**	
All patients	97	62	35	
Age (years)				0.833
≤59	48	30	18	
>59	48	31	17	
Gender				0.660
Male	61	40	21	
Female	36	22	14	
Lymph node positive				0.055
=0	47	25	22	
>0	41	30	11	
Tumor size				0.825
≤4 cm	59	38	21	
>4 cm	37	23	14	
Grade				0.007
II	66	48	18	
III	30	14	16	
IV	1	0	1	
Stage				0.303
1	37	21	16	
2	55	38	17	
4	2	1	1	
T Infiltrate				0.918
T1	3	2	1	
T2	73	46	27	
T3	20	13	7	
Lymphatic metastasis (N)				0.092
N0	50	28	22	
N1	41	30	11	
History of diabetes				0.514
No	5	4	1	
Yes	61	40	21	

**Table 3:** Relationship between EIF3B expression and tumor grade in patients with pancreatic cancer.

		**EIF3B**
Grade	Spearman correlation	0.274
	Signification (double-tailed)	0.007
	*N*	97

### Cell Models of the Knockdown of Eukaryotic Translation Initiation Factor 3 Subunit B Were Constructed

The expression levels of EIF3B in PC cell lines including PANC-1, SW1990, AsPC-1, and BxPC-3 were detected by qRT-PCR. The results showed that the PANC-1 cells expressed the highest EIF3B level, while SW1990 cells expressed the lowest EIF3B level (Figure 1B). Thus, we selected both of these cell lines with completely different expression levels of EIF3B for use in the subsequent experiments. In addition, the knockdown efficiencies of EIF3B were detected by qRT-PCR to screen the effective interference targets. The results suggested that compared with those of the shCtrl group, the knockdown efficiencies of EIF3B in the shEIF3B-1, shEIF3B-2, and shEIF3B-3 groups were 94.9% (*P* < 0.01), 86.2% (*P* < 0.05), and 92.4% (*P* < 0.01), respectively (Figure 1C). Thus, EIF3B knockdown cell models were constructed by infecting shCtrl (as negative control) and shEIF3B-1 (for silencing EIF3B). After infecting with shCtrl or shEIF3B for 72 h, the fluorescence of cells was observed by microscope, and a >80% efficiency of infection was obtained (Figure 1D). Also, the knockdown efficiencies of EIF3B in PANC-1 and SW1990 cells were 94.8% (*P* < 0.01) and 83.5% (*P* < 0.001), respectively (Figure 1E). The western blot indicated that the protein levels of EIF3B in the shEIF3B group compared with the shCtrl group were down-regulated (Figure 1F). The above results illustrated that the cell models of EIF3B knockdown were successfully constructed.

### Eukaryotic Translation Initiation Factor 3 Subunit B Knockdown Inhibited Cell Proliferation and Migration *in vitro*

**Figure 2:**
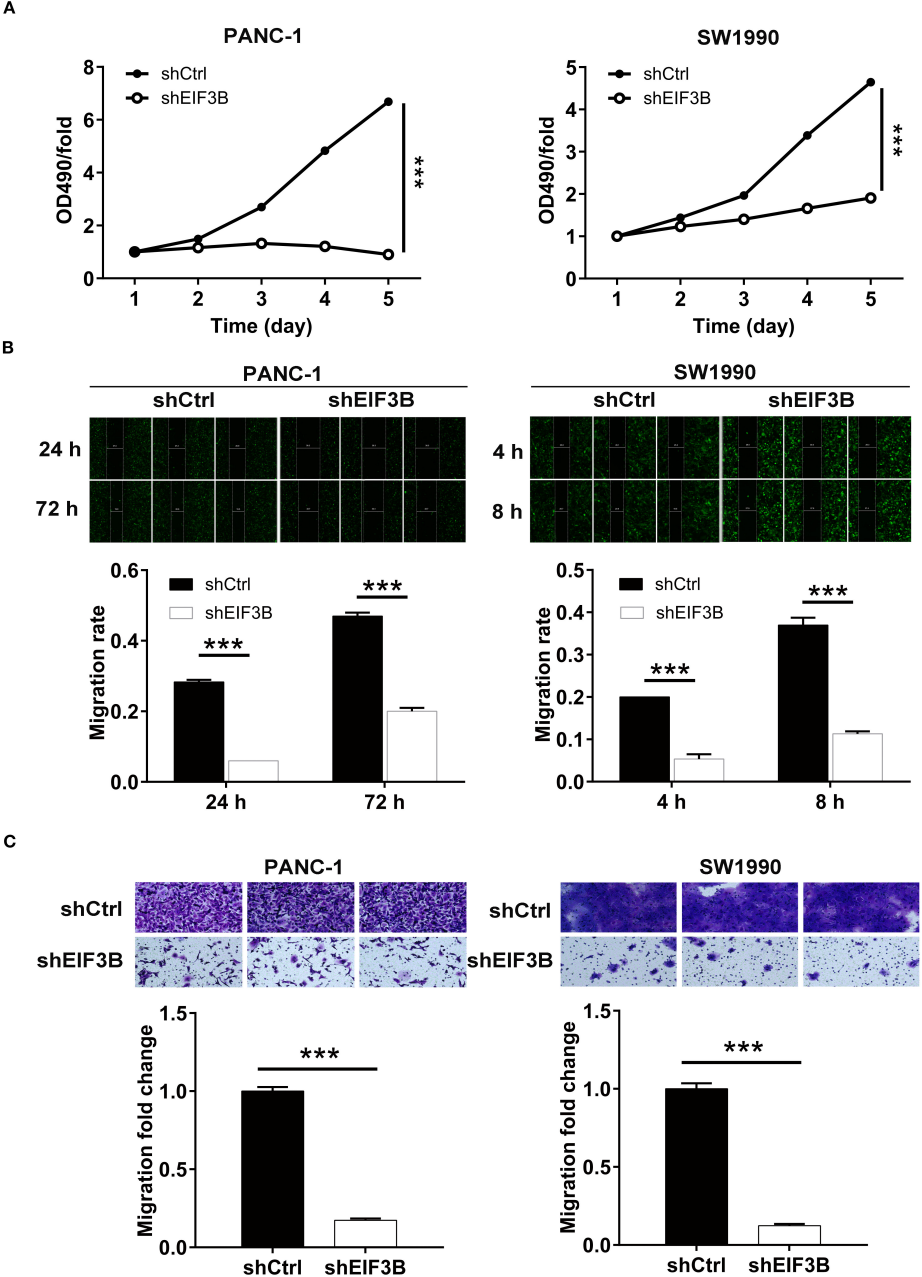
Eukaryotic translation initiation factor 3 subunit B (EIF3B) knockdown inhibited cell proliferation and migration. **(A)** The cell proliferation rate was evaluated in pancreatic cancer (PC) cell lines after infection by MTT assay. **(B)** The migration rate of cells was detected in PC cell lines after infection by wound-healing assay. **(C)** The migration rate of cells was detected in PC cell lines after infection by transwell assay. Results are presented as mean ± SD. ***P < 0.001.

The effects of EIF3B knockdown on PC cells were detected *in vitro*. The results of the MTT assay showed that the cell proliferation of the shEIF3B group was slower than that of the shCtrl group in PANC-1 and SW1990 cells, and the fold changes were −7.4 and −2.4, respectively (*P* < 0.001, Figure 2A). Then, the migration rate of cells was determined by the wound-healing assay and the transwell assay. In PANC-1 cells, the cell migration rate of the shEIF3B group was decreased by 57% in 72 h, which in SW1990 cells was decreased by 68% (8 h) (*P* < 0.001, Figure 2B). The transwell assay showed that the migration rate of cells in the shEIF3B group was decreased by 83% and 88% in PANC-1 and SW1990 cells, respectively (*P* < 0.001, Figure 2C). Collectively, the results indicated that EIF3B knockdown could inhibit the abilities of cell proliferation and migration in PC cell lines.

### The Knockdown of Eukaryotic Translation Initiation Factor 3 Subunit B Promoted Cell Apoptosis and Affected Cell Cycle

**Figure 3:**
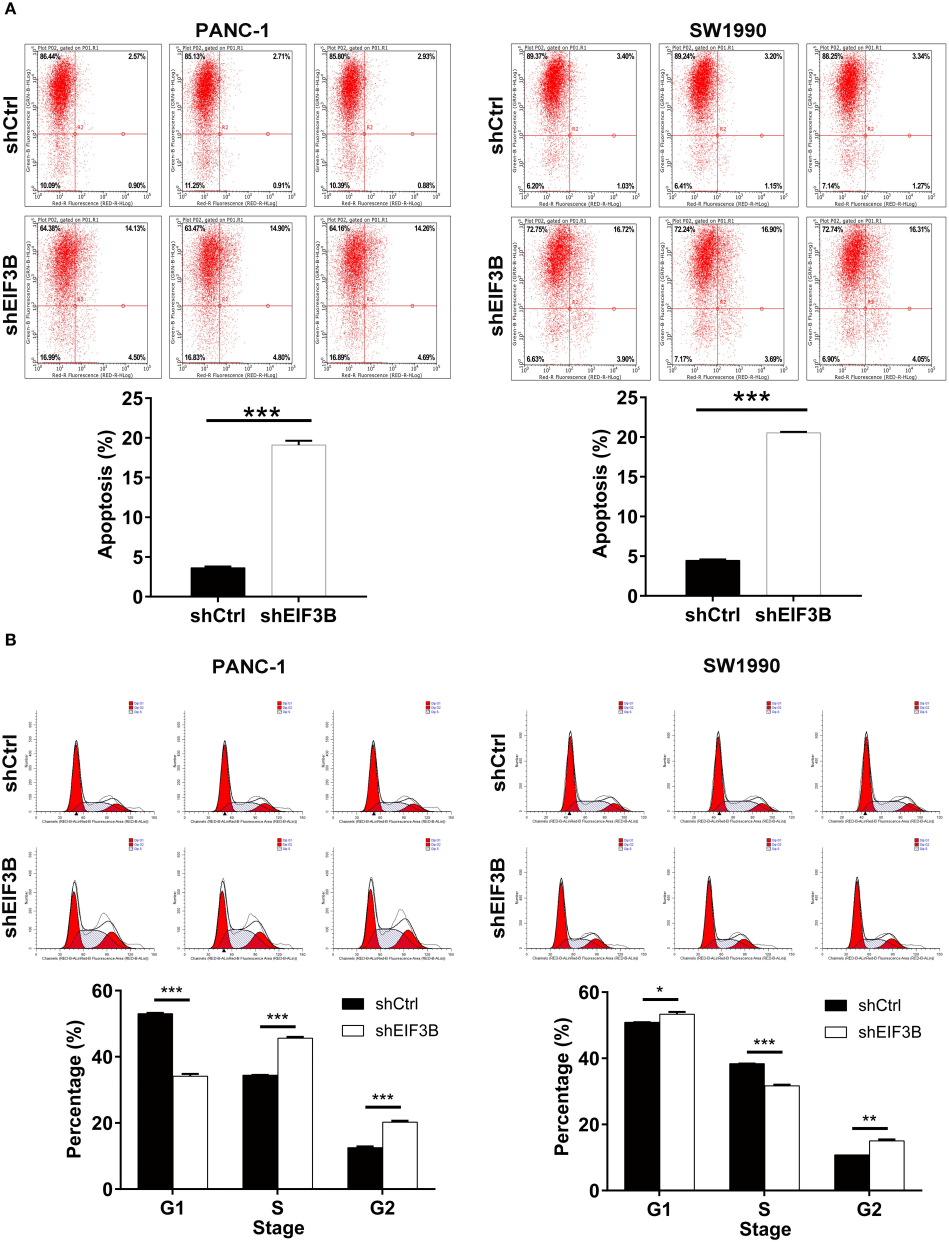
The effects of eukaryotic translation initiation factor 3 subunit B (EIF3B) knockdown on cell apoptosis and cell cycle. **(A)** The effects of EIF3B knockdown on cell apoptosis were examined by flow cytometry. **(B)** The effects of EIF3B knockdown on cell cycle were determined by flow cytometry. Results are presented as mean ± SD. *P < 0.05, **P < 0.01, ***P < 0.001.

**Figure 4:**
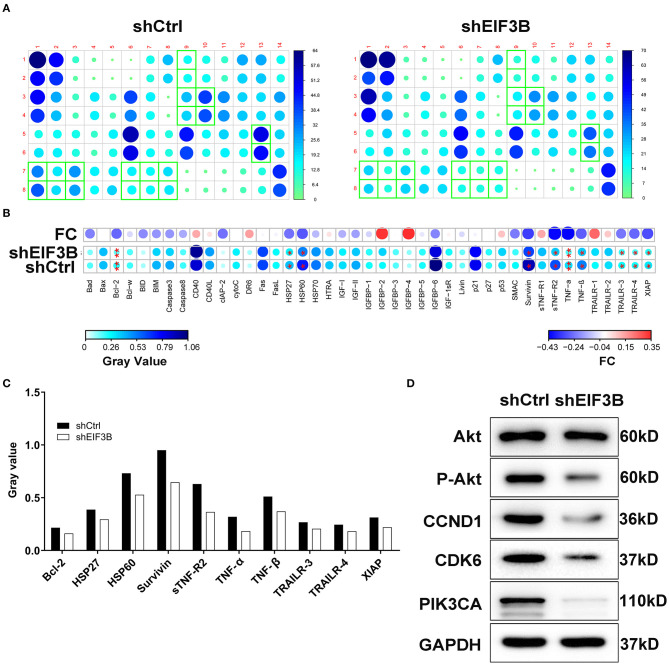
The apoptotic molecular mechanism was investigated by Human Apoptosis Antibody Array. **(A)** The expression of apoptosis-related proteins in pancreatic cancer (PC) cells infected with shEIF3B was measured by enhanced chemiluminescence (ECL) with Human Apoptosis Antibody Array. The results circled in red indicated that the protein expression was up-regulated and *P* < 0.05. **(B)** Protein expression was presented in grayscale and visualized by R studio. **(C)** The expression levels of apoptosis-related proteins were analyzed in PC cells with shEIF3B. **(D)** The expression of Akt, p-Akt, CCND1, CDK6, and PIK3CA was detected by western blot in SW1990 cells of the shCtrl and shEIF3B groups. Results are presented as mean ± SD. *P < 0.05, **P < 0.01.

After the infection of lentivirus, the effects of EIF3B knockdown on cell apoptosis and cell cycle were further examined by flow cytometry. The results showed that the ability of the cell apoptosis in the shEIF3B group increased compared with the shCtrl group, and the fold changes were 5.3 and 4.6 in PANC-1 and SW1990 cell lines, respectively (*P* < 0.001, Figure 3A). In addition, compared with the shCtrl group, the percentage of cells in the G2 phase increased in PANC-1 cells (*P* < 0.001) and SW1990 cells (*P* < 0.01, Figure 3B). Furthermore, the expression of proteins involved in apoptosis signaling pathway in SW1990 cells was detected by the Human Apoptosis Antibody Array. The results demonstrated that the knockdown of EIF3B induced the down-regulation of Bcl-2, HSP27, HSP60, Survivin, sTNF-R2, TNF-α, TNF-β, TRAILR-3, TRAILR-4, and XIAP (*P* < 0.05). It was speculated that EIF3B knockdown affected the cell apoptosis by regulating these proteins (Figures 4A–C). In addition, in SW1990 cells, the mechanism of the regulation of EIF3B knockdown in PC was initially explored. Some cancer-related factors including Akt, p-Akt, CCND1, CDK6, and PIK3CA were detected by the western blot. The results indicated that the expression of p-Akt, CCND1, CDK6, and PIK3CA in the shEIF3B group was down-regulated, and these factors could be involved in the EIF3B-associated regulation of PC (Figure 4D).

### Eukaryotic Translation Initiation Factor 3 Subunit B Knockdown Suppressed Pancreatic Cancer Growth *in vivo*

**Figure 5:**
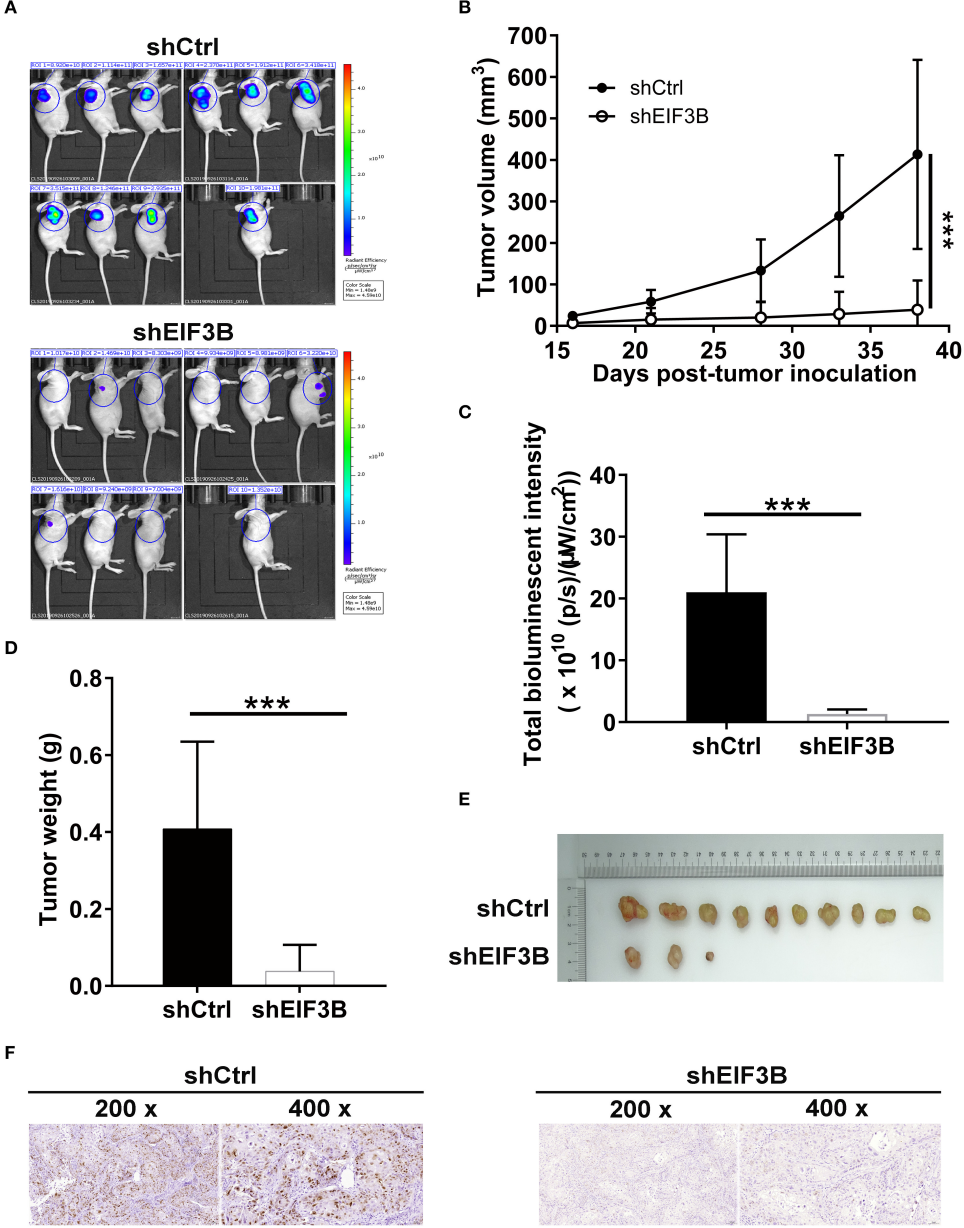
Eukaryotic translation initiation factor 3 subunit B (EIF3B) knockdown suppressed pancreatic cancer (PC) growth *in vivo*. **(A)** A nude mice model of EIF3B knockdown was constructed. **(B)** The volume of tumors was tested from feeding to sacrifice. **(C)** The fluorescence intensity was obtained through injection of D-Luciferase before sacrificing the mice. **(D)** The weight of tumors was measured after sacrificing the mice. **(E)** The photograph of tumors was taken after removing tumors. **(F)** The value of Ki-67 was detected by immunohistochemistry (IHC) in tumor sections. Results are presented as mean ± SD. ***P < 0.001.

To verify the effects of EIF3B on PC growth *in vivo*, SW1990 cells with or without EIF3B knockdown were subcutaneously injected into nude mice to construct the xenograft models (Figure 5A). The results indicated that the growth of tumors was slower in the shEIF3B group according to the tumor volume (*P* < 0.001, Figure 5B). Before sacrificing mice, the tumors were observed *in vivo* imaging, and the decreased fluorescence showed that the growth of tumors was inhibited in the shEIF3B group (*P* < 0.001, Figure 5C). Besides, after sacrificing mice, the removed tumors were graphed and weighed, and the weight of tumors was reduced (*P* < 0.05, Figures 5D,E). Moreover, the decreased Ki-67 index detected in tumor sections in SW1990 cells showed that EIF3B knockdown inhibited the expression of Ki-67 (*P* < 0.01, Figure 5F). In general, all the above results suggested that EIF3B knockdown could inhibit the tumor growth of PC *in vivo*.

## Discussion

The early diagnosis of PC with high incidence was difficult. The incidence of PC in China ranked eighth among malignant tumors, the rate of which continued to rise, and the 5-year survival rate was only 7.2% (21). Despite that the treatment strategies of PC have been constantly updating, the current efficacies of diagnosis and treatment were still not optimistic, resulting in the patient's prognosis not being significantly improved (10, 11). At present, the targeted drug therapy has made a major breakthrough and has become a promising treatment (14, 22). For example, erlotinib and gemcitabine inhibited the growth of tumor by activating Akt and nuclear factor-κB (NF-κB) signaling pathway and resulted in an obvious improvement in the survival of advanced PC patients (23). Genistein and erlotinib strengthened the effects of inhibiting EGFR/Akt/NF-κB signaling pathway on the occurrence and development of pancreas cancer (9). In this study, we reported for the first time the roles of EIF3B in the development of PC, which might be a promising therapeutic target for PC treatment.

EIF3B, a major scaffold protein, can regulate the translation initiation process of mRNA, thereby selectively regulating protein synthesis and ultimately regulating cell growth (16). The previous study showed that EIF3B expression was related to human bladder and prostate cancer prognosis, which was responsible for tumor growth and thus worked as an attractive therapeutic target (14). In addition, Tian et al. reported that the overexpression of EIF3B was correlated with the development of disease and poor prognosis, and it promoted cell proliferation and inhibited apoptosis in non-small-cell lung cancer (24). EIF3B silencing resulted in the decrease of cell viability and the increase of apoptosis level in osteosarcoma cells, and EIF3B could participate in osteosarcoma cell proliferation by targeting TNFRSF21 (25). However, the functions of EIF3B in other types of human cancers such as PC were uncertain. In this study, we found that EIF3B expression was up-regulated in PC tumor tissues. EIF3B knockdown inhibited cell proliferation and migration. At the same time, we found that upon EIF3B knockdown, SW1990 cells showed a reduction of cells in the S phase with a concomitant increase in G2/M. However, surprisingly, PANC-1 cells showed increase in both the S phase and G2/M cells with a concomitant reduction in cells in the G1 phase. We speculated that this might be related to the difference in the cell backgrounds, as SW1990 cells were isolated from the metastatic foci of patients with pancreatic lung cancer in 1978 and its doubling time was 64 h, while PANC-1 cells were derived from the malignant tumors of human ductal epithelial cells and the doubling time was 52 h. On the other hand, the previous study indicated that compared with the 0-Gy group, after continuous irradiation of ^125^I seeds, PANC-1 cells showed obvious increase in the G1, S, and G2/M phases, while SW1990 cells presented a reduction of cells in the G1 phase with increase in the S and G2/M phases. Thus, we believed that these discrepancies were reasonable (26). Also, we showed that the promotion of cell apoptosis caused by EIF3B knockdown might be achieved through regulating the apoptosis-related proteins including Bcl-2, HSP27, HSP60, Survivin, sTNF-R2, TNF-α, TNF-β, TRAILR-3, TRAILR-4, and XIAP. More importantly, we found that EIF3B knockdown made the growth of tumor slower *in vivo*. All these illustrated that EIF3B was a tumor promoter of PC.

Akt regulated cell growth, apoptosis, angiogenesis and invasion by activating NF-κB, which was reported as a target for the treatment of PC (23). In this study, we found that EIF3B knockdown made the expression of p-Akt down-regulated. CCND1 was frequently overexpressed in various kinds of human cancers as a well-known cancer-related gene (27). In PC, CCND1 was up-regulated in tumor tissues, which was significantly correlated with the degree of differentiation and poor prognosis. CCND1 also played important roles in the formation and progression of PC (28). Moreover, miR-720 could directly target CCND1 as a tumor suppressor for the treatment of PC (27). Consistently, our results showed that CCND1 was significantly down-regulated in the shEIF3B group. CDK6 was involved in the occurrence, development and metastasis of non-small-cell lung cancer by regulating cell cycle and cell proliferation (29). Similarly, our results suggested that CDK6 expression was significantly down-regulated in the shEIF3B group. Besides, PIK3CA was abundantly expressed in many human cancers, regulating cell growth, apoptosis, and proliferation (30, 31). In this study, we found that the expression of PIK3CA was down-regulated in the shEIF3B group. Ma et al. revealed that EIF3B promotes gastric cancer development at both the *in vivo* and *in vitro* levels. Besides, they revealed that the role of EIF3B in gastric cancer cell proliferation is associated with the E2F1 signaling pathway and EIF3B may affect cell proliferation by p53-independent regulation (32). We will further investigate whether EIF3B knockdown suppressed cell proliferation through E2F1 signaling pathway or p53-independent regulation in PC cells. Although the above results were obtained, more studies are needed to address the specific regulatory mechanism of EIF3B in PC.

In conclusion, the expression level of EIF3B, as reported here, was raised in PC; this meant that either the EIF3B protein itself or related signaling pathways are involved in this process. Therefore, EIF3B might be regarded as an alternative therapeutic target for more effective treatment of PC. Suppressing the expression of EIF3B or interrupting its signaling pathways may prevent or even reverse the process of tumor development. On the other hand, it was reported that EIF5A inhibition had anti-tumorigenic effects on leukemia cells when given either alone or in combination with imatinib. Besides, the EIF5A protein has been used to develop new drugs for the treatment of cancer. EIF3B as a promising therapeutic target may have similar effects to EIF5A in cancer treatment.

## Data Availability Statement

The raw data supporting the conclusions of this article will be made available by the authors, without undue reservation.

## Ethics Statement

The animal studies were reviewed and approved by the Medical Ethics committee of People's Hospital of Deyang City. The studies involving human participants were reviewed and approved by the Medical Ethics committee of People's Hospital of Deyang City. Written informed consent to participate in this study was provided by the patients.

## Author Contributions

HR and WS designed this research. GM and CY operated the cell and animal experiments. YL and RX conducted the data procession and analysis. HR completed the manuscript, which was reviewed by WS. All the authors have confirmed the submission of this manuscript.

## Conflict of Interest

The authors declare that the research was conducted in the absence of any commercial or financial relationships that could be construed as a potential conflict of interest.
